# Supplementary material for: Optimized vectors for genetic engineering of Aureobasidium pullulans
Source: bioRxiv. 2025 Jan 27:2025.01.25.634885. Preprint. [Version 1] doi: 10.1101/2025.01.25.634885 (PMC11838232; doi:10.1101/2025.01.25.634885)
Supplement: Supplement 1 [file media-1.pdf]

Supplemental Table 1

Strain name	Background	Relevant Genotype	Strain description
DLY23540	EXF-150		WT *A. pullulans*
DLY24148	EXF-150	*ura3Δ::HYG*^{R}	*A. pullulans* uracil auxotroph with a hygromycin resistance cassette replacing *URA3*
DLY25289	EXF-150	*CIT1-GFP : NAT*^{R}	*A. pullulans* expressing *CIT1* C-terminally tagged with GFP using Nat selection
DLY25290	EXF-150	*CIT1-GFP : NAT*^{R}	*A. pullulans* expressing *CIT1* C-terminally tagged with GFP using Nat selection
DLY25291	EXF-150	*CIT1-GFP : NAT*^{R}	*A. pullulans* expressing *CIT1* C-terminally tagged with GFP using Nat selection
DLY25292	EXF-150	*CIT1-mCherry : NAT*^{R}	*A. pullulans* expressing *CIT1* C-terminally tagged with mCherry using Nat selection
DLY25293	EXF-150	*CIT1-mCherry : NAT*^{R}	*A. pullulans* expressing *CIT1* C-terminally tagged with mCherry using Nat selection
DLY25294	EXF-150	*CIT1-mCherry : NAT*^{R}	*A. pullulans* expressing *CIT1* C-terminally tagged with mCherry using Nat selection
DLY25295	EXF-150	*CIT1-sfGFP : NAT*^{R}	*A. pullulans* expressing *CIT1* C-terminally tagged with sfGFP using Nat selection
DLY25297	EXF-150	*CIT1-sfGFP : NAT*^{R}	*A. pullulans* expressing *CIT1* C-terminally tagged with sfGFP using Nat selection
DLY25298	EXF-150	*CIT1-mStayGold : NAT*^{R}	*A. pullulans* expressing *CIT1* C-terminally tagged with mStayGold using Nat selection
DLY25299	EXF-150	*CIT1-mStayGold : NAT*^{R}	*A. pullulans* expressing *CIT1* C-terminally tagged with mStayGold using Nat selection
DLY25300	EXF-150	*CIT1-mStayGold : NAT*^{R}	*A. pullulans* expressing *CIT1* C-terminally tagged with mStayGold using Nat selection
DLY25301	EXF-150	*CIT1-Dendra2 : NAT*^{R}	*A. pullulans* expressing *CIT1* C-terminally tagged with Dendra2 using Nat selection
DLY25302	EXF-150	*CIT1-Dendra2 : NAT*^{R}	*A. pullulans* expressing *CIT1* C-terminally tagged with Dendra2 using Nat selection
DLY25303	EXF-150	*CIT1-Dendra2 : NAT*^{R}	*A. pullulans* expressing *CIT1* C-terminally tagged with Dendra2 using Nat selection
DLY25343	EXF-150	*CIT1-mNeon : NAT*^{R}	*A. pullulans* expressing *CIT1* C-terminally tagged with mNeon using Nat selection
DLY25344	EXF-150	*CIT1-mNeon : NAT*^{R}	*A. pullulans* expressing *CIT1* C-terminally tagged with mNeon using Nat selection
DLY25345	EXF-150	*CIT1-mNeon : NAT*^{R}	*A. pullulans* expressing *CIT1* C-terminally tagged with mNeon using Nat selection
DLY25346	EXF-150	*CIT1-mScarlet : NAT*^{R}	*A. pullulans* expressing *CIT1* C-terminally tagged with mScarlet using Nat selection
DLY25347	EXF-150	*CIT1-mScarlet : NAT*^{R}	*A. pullulans* expressing *CIT1* C-terminally tagged with mScarlet using Nat selection
DLY25348	EXF-150	*CIT1-mScarlet : NAT*^{R}	*A. pullulans* expressing *CIT1* C-terminally tagged with mScarlet using Nat selection
DLY25619	EXF-150	*URA3 : SpH2Bp-3xmCherry : ScACT1p-3xGFP*	*A. pullulans* expressing cytoplasmic 3xmCherry under strong SpH2B promoter and cytoplasmic 3xGFP under ScACT1 promoter integrated at the *URA3* locus next to *URA*[illegible]
DLY25620	EXF-150	*URA3 : SpH2Bp-3xmCherry : ScACT1p-3xGFP*	*A. pullulans* expressing cytoplasmic 3xmCherry under strong SpH2B promoter and cytoplasmic 3xGFP under ScACT1 promoter integrated at the *URA3* locus next to *URA*[illegible]
DLY25621	EXF-150	*URA3 : SpH2Bp-3xmCherry : ScACT1p-3xGFP*	*A. pullulans* expressing cytoplasmic 3xmCherry under strong SpH2B promoter and cytoplasmic 3xGFP under ScACT1 promoter integrated at the *URA3* locus next to *URA*[illegible]
DLY25625	EXF-150	*URA3 : SpH2Bp-3xGFP : SpH2Ap-3xmCherry*	*A. pullulans* expressing cytoplasmic 3xmCherry under strong SpH2A promoter and cytoplasmic 3xGFP under SpH2B promoter integrated at the *URA3* locus next to *URA*[illegible]
DLY25626	EXF-150	*URA3 : SpH2Bp-3xGFP : SpH2Ap-3xmCherry*	*A. pullulans* expressing cytoplasmic 3xmCherry under strong SpH2A promoter and cytoplasmic 3xGFP under SpH2B promoter integrated at the *URA3* locus next to *URA*[illegible]
DLY25627	EXF-150	*URA3 : SpH2Bp-3xGFP : SpH2Ap-3xmCherry*	*A. pullulans* expressing cytoplasmic 3xmCherry under strong SpH2A promoter and cytoplasmic 3xGFP under SpH2B promoter integrated at the *URA3* locus next to *URA*[illegible]
DLY25628	EXF-150	*URA3 : ScACT1p-3xmCherry : ApACT1p-3xGFP*	*A. pullulans* expressing cytoplasmic 3xmCherry under ScACT1 promoter and cytoplasmic 3xGFP under ApACT1 promoter integrated at the *URA3* locus next to *URA3*
DLY25629	EXF-150	*URA3 : ScACT1p-3xmCherry : ApACT1p-3xGFP*	*A. pullulans* expressing cytoplasmic 3xmCherry under ScACT1 promoter and cytoplasmic 3xGFP under ApACT1 promoter integrated at the *URA3* locus next to *URA3*
DLY25630	EXF-150	*URA3 : ScACT1p-3xmCherry : ApACT1p-3xGFP*	*A. pullulans* expressing cytoplasmic 3xmCherry under ScACT1 promoter and cytoplasmic 3xGFP under ApACT1 promoter integrated at the *URA3* locus next to *URA3*
DLY25631	EXF-150	*URA3 : ScACT1p-3xmCherry : ApTUB1p-3xGFP*	*A. pullulans* expressing cytoplasmic 3xmCherry under ScACT1 promoter and cytoplasmic 3xGFP under ApTUB1 promoter integrated at the *URA3* locus next to *URA3*
DLY25632	EXF-150	*URA3 : ScACT1p-3xmCherry : ApTUB1p-3xGFP*	*A. pullulans* expressing cytoplasmic 3xmCherry under ScACT1 promoter and cytoplasmic 3xGFP under ApTUB1 promoter integrated at the *URA3* locus next to *URA3*
DLY25633	EXF-150	*URA3 : ScACT1p-3xmCherry : ApTUB1p-3xGFP*	*A. pullulans* expressing cytoplasmic 3xmCherry under ScACT1 promoter and cytoplasmic 3xGFP under ApTUB1 promoter integrated at the *URA3* locus next to *URA3*
DLY24594	EXF-150	*leu2Δ::NAT*^{R}	*A. pullulans* uracil auxotroph with a nourseothricin resistance cassette replacing *LEU2*
DLY24595	EXF-150	*leu2Δ::NAT*^{R}	*A. pullulans* uracil auxotroph with a nourseothricin resistance cassette replacing *LEU2*
DLY24596	EXF-150	*leu2Δ::NAT*^{R}	*A. pullulans* uracil auxotroph with a nourseothricin resistance cassette replacing *LEU2*
DLY25507	EXF-150	*leu2Δ::HYG*^{R}	*A. pullulans* uracil auxotroph with a hygromycin resistance cassette replacing *LEU2*
DLY25508	EXF-150	*leu2Δ::HYG*^{R}	*A. pullulans* uracil auxotroph with a hygromycin resistance cassette replacing *LEU2*
DLY25509	EXF-150	*leu2Δ::HYG*^{R}	*A. pullulans* uracil auxotroph with a hygromycin resistance cassette replacing *LEU2*
DLY25510	EXF-150	*leu2Δ::G418*^{R}	*A. pullulans* uracil auxotroph with a geneticin resistance cassette replacing *LEU2*
DLY25511	EXF-150	*leu2Δ::G418*^{R}	*A. pullulans* uracil auxotroph with a geneticin resistance cassette replacing *LEU2*
DLY25512	EXF-150	*leu2Δ::G418*^{R}	*A. pullulans* uracil auxotroph with a geneticin resistance cassette replacing *LEU2*
