# Supplementary material for: Optimized vectors for genetic engineering of Aureobasidium pullulans
Source: bioRxiv. 2025 Jan 27:2025.01.25.634885. Preprint. [Version 1] doi: 10.1101/2025.01.25.634885 (PMC11838232; doi:10.1101/2025.01.25.634885)
Supplement: Supplement 2 [file media-2.pdf]

Supplemental Table 2

Plasmid name	Original ID#	Plasmid description
pAP-U2-1	4798	Plasmid for integration at the native *URA3* locus with 3xmCherry and 3xGFP under control of the strong bidirectional SpH2A/B promoter.
pAP-U2-2	4856	Plasmid for integration at the native *URA3* locus with 3xmCherry under control of the SpH2B promoter and 3xGFP under control of the ScACT1 promoter.
pAP-U2-3	4826	Plasmid for integration at the native *URA3* locus with 3xmCherry under control of the ScACT1 promoter and 3xGFP under control of the ApACT1 promoter.
pAP-U2-4	4883	Plasmid for integration at the native URA3 locus with 3xmCherry under control of the ScACT1 promoter and 3xGFP under control of the ApTUB1 promoter.
pAPInt-GFP-NatR	4760	Plasmid for deleting or C-terminally tagging endogenous genes with GFP and selection on Nat.
pAPInt-GFP-HygR	4766	Plasmid for deleting or C-terminally tagging endogenous genes with GFP and selection on Hyg.
pAPInt-GFP-G418F	4946	Plasmid for deleting or C-terminally tagging endogenous genes with GFP and selection on G418.
pAPInt-sfGFP-NatI	4953	Plasmid for deleting or C-terminally tagging endogenous genes with sfGFP and selection on Nat.
pAPInt-sfGFP-HygI	4905	Plasmid for deleting or C-terminally tagging endogenous genes with sfGFP and selection on Hyg.
pAPInt-sfGFP-G41	4948	Plasmid for deleting or C-terminally tagging endogenous genes with sfGFP and selection on G418.
pAPInt-mNeonGre	4908	Plasmid for deleting or C-terminally tagging endogenous genes with mNeonGreen and selection on Nat.
pAPInt-mNeonGre	4909	Plasmid for deleting or C-terminally tagging endogenous genes with mNeonGreen and selection on Hyg.
pAPInt-mNeonGre	4951	Plasmid for deleting or C-terminally tagging endogenous genes with mNeonGreen and selection on G418.
pAPInt-mStayGold	4901	Plasmid for deleting or C-terminally tagging endogenous genes with mStayGold and selection on Nat.
pAPInt-mStayGold	4904	Plasmid for deleting or C-terminally tagging endogenous genes with mStayGold and selection on Hyg.
pAPInt-mStayGold	4949	Plasmid for deleting or C-terminally tagging endogenous genes with mStayGold and selection on G418.
pAPInt-mCherry-N	4767	Plasmid for deleting or C-terminally tagging endogenous genes with mCherry and selection on Nat.
pAPInt-mCherry-H	4768	Plasmid for deleting or C-terminally tagging endogenous genes with mCherry and selection on Hyg.
pAPInt-mCherry-G	4947	Plasmid for deleting or C-terminally tagging endogenous genes with mCherry and selection on G418.
pAPInt-mScarlet-N	4911	Plasmid for deleting or C-terminally tagging endogenous genes with mScarlet and selection on Nat.
pAPInt-mScarlet-H	4900	Plasmid for deleting or C-terminally tagging endogenous genes with mScarlet and selection on Hyg.
pAPInt-mScarlet-G	4952	Plasmid for deleting or C-terminally tagging endogenous genes with mScarlet and selection on G418.
pAPInt-Dendra2-N	4903	Plasmid for deleting or C-terminally tagging endogenous genes with mDendra2 and selection on Nat.
pAPInt-Dendra2-H	4906	Plasmid for deleting or C-terminally tagging endogenous genes with mDendra2 and selection on Hyg.
pAPInt-Dendra2-G	4950	Plasmid for deleting or C-terminally tagging endogenous genes with mDendra2 and selection on G418.
