# Supplementary material for: Optimized vectors for genetic engineering of Aureobasidium pullulans
Source: bioRxiv. 2025 Jan 27:2025.01.25.634885. Preprint. [Version 1] doi: 10.1101/2025.01.25.634885 (PMC11838232; doi:10.1101/2025.01.25.634885)
Supplement: Supplement 3 [file media-3.pdf]

Supplemental Table 3

Primer name	Sequence	Primer description
apLEU2_pAPint_DEL_F	ACCATCTACACACAACACACACACATTACTACACTTGGTGCGGGTGAAAGGTCTTGG	Primers used in 3-part-PCR to delete *LEU2*
apLEU2_pAPint_DEL_R	CATATCCTTGCGTAGCTTTTTGCGTATCCTCTCTTCAGTATAGCGACCAGCATTCACATACG	Primers used in 3-part-PCR to delete *LEU2*
apLEU2_ups_F	GTCAGGTCATTCCGCAGGTGTGTAG	Primers used in 3-part-PCR to delete *LEU2*
apLEU2_ups_R	CTTTGCGACTTGACCAAGACCTTTCACCCGCACCAAGTGTAGTAATGTGTGTGTGTTGTGTG	Primers used in 3-part-PCR to delete *LEU2*
apLEU2_dwn_F	GCGTCAATCGTATGTGAATGCTGGTCGCTATACTGAAGAGAGGATACGCAAAAAGCTACG	Primers used in 3-part-PCR to delete *LEU2*
apLEU2_dwn_R	GCTATTGTCTGCGTGCCAGTGC	Primers used in 3-part-PCR to delete *LEU2*
apCit1_ups_F	GTTCGTTGAGGAGCTCATCGACC	Primers used in 3-part-PCR to tag *CIT1*
apCit1_ups_R	catagaaccagaaccagcaccgtcaccAAGCTTAGCACCAACAAGCTTGG	Primers used in 3-part-PCR to tag *CIT1*
apCit1_pAPint_F	GACGCCTGGGCCAAGCTTGTTGGTGCTAAGCTTggtgacggtgctggttctgg	Primers used in 3-part-PCR to tag *CIT1*
apCit1_pAPint_R	GATAGACCAAAGTCTACTCCCAATCTTCAGcgcataggccactagtggatctg	Primers used in 3-part-PCR to tag *CIT1*
apCit1_dwn_F	tatactgcagatccactagtggcctatgcgCTGAAGATTGGGAGTAGACTTTGGTC	Primers used in 3-part-PCR to tag *CIT1*
apCit1_dwn_R	GACAGGCTTCTTCTCGAGCTTGG	Primers used in 3-part-PCR to tag *CIT1*
