# Supplementary material for: Optimized vectors for genetic engineering of Aureobasidium pullulans
Source: bioRxiv. 2025 Jan 27:2025.01.25.634885. Preprint. [Version 1] doi: 10.1101/2025.01.25.634885 (PMC11838232; doi:10.1101/2025.01.25.634885)

bioRxiv preprint doi: https://doi.org/10.1101/2025.01.25.634885; this version posted January 27, 2025. The copyright holder for this preprint (which was not certified by peer review) is the author/funder, who has granted bioRxiv a license to display the preprint in perpetuity. It is made available under aCC-BY 4.0 International license.

SUPPLEMENTAL FIGURE CAPTIONS

Supplemental Figure 1: Determination of the minimal inhibitory G418 concentration. (A) Number of colonies present on plates of varying G418 concentrations from two separate experiments. (B) Comparison of plates without G418 and with the concentration used for selection.

Supplemental Figure 2: Fluorescence decay curves for each fluorophore. (A-B) Fluorescence decay over time for cells expressing Cit1 tagged with the indicated red fluorescent protein (mScarlet, mCherry, or Dendra2 after photoconversion: A), or green fluorescent protein (mNG, GFP, sfGFP, or mStayGold: B). The mean and standard deviation across 15 cells is shown for each as well as the one phase decay curve fit to each dataset. The R squared values for each curve fitting are 0.75, 0.86, 0.76, 0.92, 0.83, 0.95, 0.78, and 0.90 for mScarlet, mCherry, Dendra2 after, mNG, GFP, sfGFP, and mStayGold, respectively.

bioRxiv preprint doi: https://doi.org/10.1101/2025.01.25.634885; this version posted January 27, 2025. The copyright holder for this preprint (which was not certified by peer review) is the author/funder, who has granted bioRxiv a license to display the preprint in perpetuity. It is made available under aCC-BY 4.0 International license.

Supplemental Figure 1

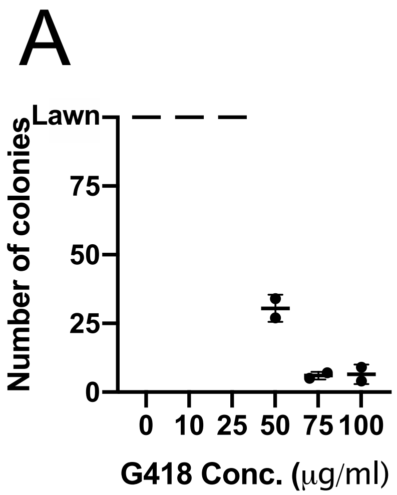

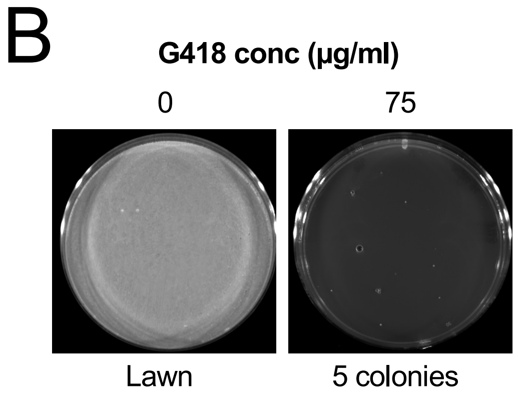

bioRxiv preprint doi: https://doi.org/10.1101/2025.01.25.634885; this version posted January 27, 2025. The copyright holder for this preprint (which was not certified by peer review) is the author/funder, who has granted bioRxiv a license to display the preprint in perpetuity. It is made available under aCC-BY 4.0 International license.

Supplemental Figure 2

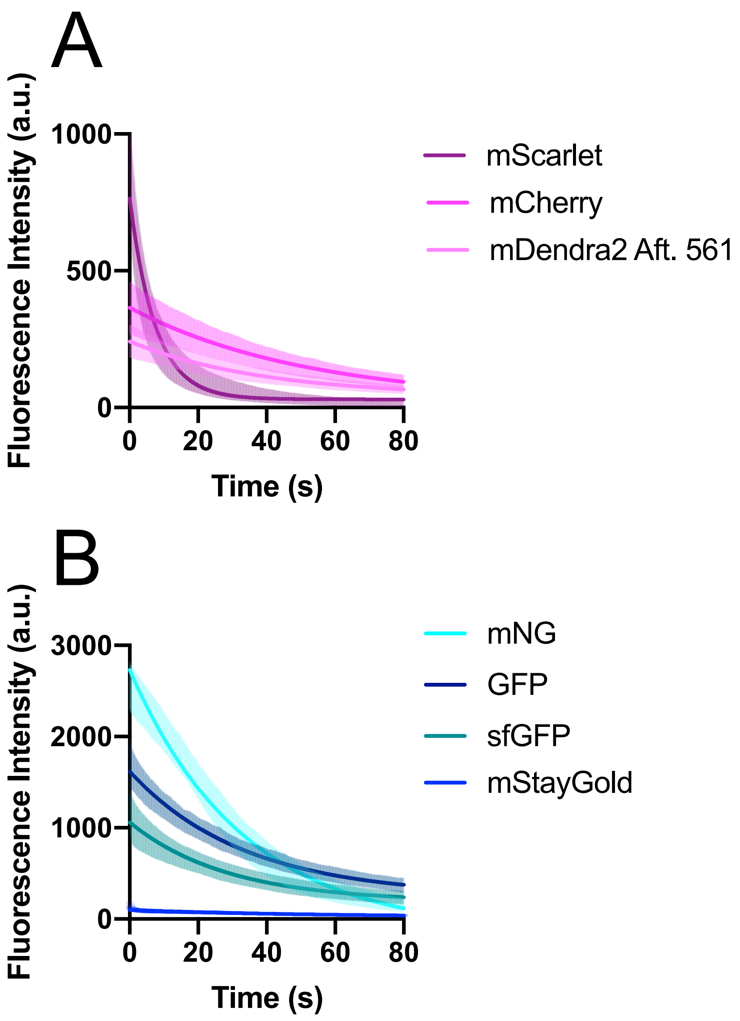

Supplement: 1 [file NIHPP2025.01.25.634885V1-supplement-1.pdf]
